# Histochemical Characterization of the Dorsal Raphe-Periaqueductal Grey Dopamine Transporter Neurons Projecting to the Extended Amygdala

**DOI:** 10.1523/ENEURO.0121-22.2022

**Published:** 2022-05-27

**Authors:** Qin Zhao, Tetsufumi Ito, Chika Soko, Yoshie Hori, Takafumi Furuyama, Hiroyuki Hioki, Kohtarou Konno, Miwako Yamasaki, Masahiko Watanabe, Satoshi Ohtsuka, Munenori Ono, Nobuo Kato, Ryo Yamamoto

**Affiliations:** 1 Department of Physiology, Kanazawa Medical University, Uchinada, Ishikawa 920-0293, Japan; 2 Systems Function and Morphology Laboratory, Graduate School of Innovative Life Science, University of Toyama, Toyama, Toayama 930-0194, Japan; 3 Department of Neuroanatomy, Juntendo University Graduate School of Medicine, Tokyo, 113-8421, Japan; 4 Department of Anatomy, Faculty of Medicine, Hokkaido University, Sapporo, Hokkaido 060-8638, Japan; 5 Laboratory for Experimental Animals, Kyoto Prefectural University of Medicine, Kyoto, Kyoto 602-8566, Japan; 6 Department of Neurology, Tongji Hospital, Tongji Medical College, Huazhong University of Science and Technology, Wuhan 430030, China

**Keywords:** amygdala, bed nucleus of the stria terminalis, dopamine, dopamine transporter, dorsal raphe, vasoactive intestinal peptide

## Abstract

The dorsal raphe (DR) nucleus contains many tyrosine hydroxylase (TH)-positive neurons which are regarded as dopaminergic (DA) neurons. These DA neurons in the DR and periaqueductal gray (PAG) region (DA^DR-PAG^ neurons) are a subgroup of the A10 cluster, which is known to be heterogeneous. This DA population projects to the central nucleus of the amygdala (CeA) and the bed nucleus of the stria terminalis (BNST) and has been reported to modulate various affective behaviors. To characterize, the histochemical features of DA^DR-PAG^ neurons projecting to the CeA and BNST in mice, the current study combined retrograde labeling with Fluoro-Gold (FG) and histological techniques, focusing on TH, dopamine transporter (DAT), vasoactive intestinal peptide (VIP), and vesicular glutamate transporter 2 (VGlut2). To identify putative DA neurons, DAT-Cre::Ai14 mice were used. It was observed that DAT^DR-PAG^ neurons consisted of the following two subpopulations: TH+/VIP– and TH–/VIP+ neurons. The DAT+/TH–/VIP+ subpopulation would be non-DA noncanonical DAT neurons. Anterograde labeling of DAT^DR-PAG^ neurons with AAV in DAT-Cre mice revealed that the fibers exclusively innervated the lateral part of the CeA and the oval nucleus of the BNST. Retrograde labeling with FG injections into the CeA or BNST revealed that the two subpopulations similarly innervated these regions. Furthermore, using VGlut2-Cre::Ai14 mice, it was turned out that the TH–/VIP+ subpopulations innervating both CeA and BNST were VGlut2-positive neurons. These two subpopulations of DAT^DR-PAG^ neurons, TH+/VIP– and TH–/VIP+, might differentially interfere with the extended amygdala, thereby modulating affective behaviors.

## Significance Statement

Dopaminergic (DA) neurons in the dorsal raphe (DR) and periaqueductal gray (PAG) regions have projections to the extended amygdala and have been reported to modulate various affective behaviors. These DA neurons are a subgroup of the A10 cluster, which is known to be heterogeneous. However, it remains unknown how heterogeneous subpopulations innervate the extended amygdala. We used the DA transporter (DAT) as a DA neuron marker and found that the DAT^DR-PAG^ neurons are composed of at least two subpopulations, DAT+/tyrosine hydroxylase (TH)+/vasoactive intestinal peptide (VIP)– putative DA neurons and DAT+/TH–/VIP+ putative non-DA glutamatergic neurons, innervating the extended amygdala similarly. These results indicate that the two subpopulations might differently modulate the affective behaviors controlled by the extended amygdala.

## Introduction

The dorsal raphe nucleus (DR) has been recognized as a typical serotonergic (5-HT) nucleus in mammals (Takeuchi et al., 1982a, b; Ishimura et al., 1988). To date, diverse lines of studies have focused on the physiological roles of 5-HT neurons (Hornung, 2003; Muller and Jacobs, 2010; Ren et al., 2018). However, the DR not only consists of 5-HT neurons but contains also many tyrosine hydroxylase (TH) positive neurons in rodents (Trulson et al., 1985; Descarries et al., 1986; Hasue and Shammah-Lagnado, 2002; Hioki et al., 2010; Poulin et al., 2014) and primates (Arsenault et al., 1988; Charara and Parent, 1998). Recently, the functional importance of dopaminergic (DA) neurons on affective behaviors in the DR and ventral periaqueductal gray (PAG) regions has been reported in multiple studies in mice. DA neurons in the DR-PAG region (DA^DR-PAG^) influence pain sensation (Li et al., 2016; Yu et al., 2021), modulate social interactions (Matthews et al., 2016), accelerate fear learning (Groessl et al., 2018), respond to salient stimuli (Cho et al., 2017, 2021), and control memory related to reward incentives (Lin et al., 2020, 2021). These studies pointed to the bed nucleus of the stria terminalis (BNST) and the central nucleus of the amygdala (CeA), which are known to play significant roles in affective behaviors, as downstream targets of DA^DR-PAG^ neurons. Projections from TH neurons in the DR to these regions have been confirmed using a retrograde tracer (Hasue and Shammah-Lagnado, 2002).

However, a further complication is that the DA transporter (DAT)-positive neurons in the DR-PAG region (DAT^DR-PAG^ neurons), which are expected to be DA neurons, appear to have an array of diversity in colocalization with other neuron markers. At least mRNAs encoding vasoactive intestinal peptide (VIP), vesicular glutamate transporter 2 (VGlut2), calbindin, cholecystokinin, and TH are known to be expressed in these neurons (Poulin et al., 2014; La Manno et al., 2016; Hook et al., 2018; Tiklová et al., 2019; for review, see Poulin et al., 2020). This heterogeneity raises the possibility that each population of DAT^DR-PAG^ neurons plays a unique role in affective behaviors, while fine features of connections from the heterogeneous DA^DR-PAG^ neurons to the CeA and BNST remain unclear. Also, the presence of mRNA does not necessarily ensure that the encoded protein is expressed sufficiently to a physiologically relevant extent, suggesting that the confirmation of actual protein expression may also be important in evaluating heterogeneity.

Therefore, in the present study, we aimed to clarify the potential heterogeneity of DA^DR-PAG^ neurons projecting to the CeA and BNST. We applied immunohistochemistry and tracer methods focusing on DAT, TH, VIP, and VGlut2 in DAT-Cre mice (Bäckman et al., 2006), in which expression of Cre-recombinase is more restricted to DA neurons than in TH-Cre mice (Savitt et al., 2005; Lammel et al., 2015; Stuber et al., 2015). The present experiments thus showed that the majority of DAT neurons projecting onto the CeA and BNST consist of two different subpopulations. These subpopulations of DAT^DR-PAG^ neurons might play different roles in the regulation of affective behaviors.

## Materials and Methods

Experiments were performed in accordance with the guiding principles of the Physiologic Society of Japan and were approved by the Animal Care Committee of Kanazawa Medical University and University of Toyama.

### Subjects

Mice were group housed (two to five animals per cage) in a colony under a 12/12 h light/dark cycle. Food and water were available *ad libitum*. C57BL/6 (*n* = 10), DAT-ires-cre (*n* = 4), DAT-ires-Cre::Ai14 (*n* = 17), and VGlut2-ires-Cre::Ai14 (*n* = 9) were used in this study. All mice were two to five months old male. In this study, we focused on the histochemical character of males. DAT-ires-Cre mice (stock #006660; Bäckman et al., 2006) and Ai14 mice (stock #007908; Madisen et al., 2010) were purchased from The Jackson Laboratory. VGlut2-ires-Cre mice (Vong et al., 2011) were provided by Dr. Brad Lowell (Beth Israel Deaconess Medical Center, Harvard Medical School).

### Surgeries

Subjects were anesthetized with isoflurane (1–2%) during the surgery. Fluoro-Gold (FG; 3% in 0.1 m cacodylate buffer solution; Fluochrome, CO, USA) was iontophoretically applied to the CeA (in mm; AP: −1.5, ML: +2.8, from bregma; DV: −4.4, from the brain surface) or the BNST (in mm; AP: +0.2, ML: +0.7, from bregma; DV: −3.2, from the brain surface) with a 3-μA current for 6–10 min with a 50% duty cycle (2.5 s on/2.5 s off) through a sharp grass pipette. AAV5-EF1α-DIO-hChR2-EYFP (200 nl; catalog #20298-AAV5, Addgene, MA, USA) was injected into the DR-PAG region (in mm; AP: −4.3, ML: +1.0, from bregma; DV: −2.7 angled at 22°, from the brain surface) using a microinjector (Nanoject II, Drummond Scientific Company, PA, USA). 6-Hydroxydopamine hydrobromide (6-OHDA; 10 μg/μl in PBS containing 0.1% ascorbate; 200 nl ×2; Sigma-Aldrich, MO, USA) was injected into two sites of the DR-PAG region (in mm; AP: −4.1 and −4.4, ML: +1.0, from bregma; DV: −2.7 angled 22°, from the brain surface) using the microinjector (Nanoject II).

### Immunohistochemistry

After surgery, the animals were maintained in home cages. The survival period was 10 d for FG or 6-OHDA-injected mice and 14–21 d for AAV-injected mice. The subjects were deeply anesthetized with isoflurane and were perfused transcardially with 4% paraformaldehyde in 0.1 m PB (pH 7.4). Brains were dissected out and immersed in the same fixative overnight and were cryoprotected with 30% sucrose in 0.1 m PB (pH 7.4) for 2 d. Then, frozen coronal sections were cut to a thickness of 40 μm using a microtome (Yamato Koki, Saitama, Japan) equipped with a freezing stage. Every sixth section was used for histology. For fluorescent immunohistochemistry, sections were incubated overnight in some combinations of rabbit anti-FG (1:2000; AB153-I, Merck-Millipore, MA, USA), rat anti-RFP (1:1000; 5F8, Chromotek, Planegg-Martinsried, Germany), rat anti-DAT (1:500; MAB369, Merck-Millipore), mouse anti-TH (1:1000, MAB318, Merck-Millipore), guinea pig anti-VIP (1:200; Hioki et al., 2018), and rat anti-GFP antibodies (1:500; 04404, Nacalai Tesque, Kyoto, Japan) diluted in incubation buffer, which was composed of 1% normal donkey serum, PBS, 0.3% Triton X-100, and 0.02% sodium azide. On the second day, sections were washed and incubated for 2 h with secondary antibodies raised from donkey conjugated with Alexa Fluor 488, 594, 647, or Cy5 (1:200; Jackson ImmunoResearch, PA, USA). In some cases, propidium iodide (PI; 1:2000) was added in secondary antibody solution to label the Nissl substance. For FG signals, tyramide signal amplification (TSA) was applied. For the TSA reaction, a biotinylated anti-rabbit secondary antibody was used instead of a fluorescent secondary antibody. The sections were then incubated with avidin-biotinylated horseradish peroxidase complex (1:50, ABC Elite, Vector Laboratories, CA, USA) diluted in PBS with 0.3% Triton X-100. The sections were processed with biotinylated-TSA (Furuta et al., 2009; Kuramoto et al., 2009), and then incubated with Alexa Fluor 488-conjugated streptavidin (1:400, S11223, Invitrogen, MA, USA). Sections were mounted on coated slides, air-dried, rehydrated, and coverslipped with ProLong Glass (Thermo Fisher Scientific, MA, USA). The combinations of primary and secondary antibodies or TSA in each experiment are shown in Table 1. For the brightfield immunohistochemistry, sections were incubated overnight with one of the primary antibodies, washed, and incubated for 2 h with biotinylated secondary antibodies that were raised from donkey (1:200; Jackson ImmunoResearch). Then, sections were washed and incubated for 2 h with ABC (Vector). After this step, the bound peroxidase was visualized using a nickel diaminobenzidine (DAB) reaction. Sections were mounted on coated slides, air-dried, dehydrated, and coverslipped with Entellan (Merck-Millipore).

**Table 1:** Combinations of antibodies in each figure showing fluorescence images are shown in the table

Figures	Combinations
Figure 1	TH: mouse anti-TH-antibody + anti-mouse-IgG-antibody with Alexa Fluor 488 DAT (tdTomato): rat anti-RFP-antibody + anti-rat-IgG-antibody with Alexa Fluor 594 VIP: guinea pig anti-VIP-antibody + anti-guinea pig-IgG-antibody with Cy5
Figure 2	DAT: *in situ* hybridization (Fast Red TR) VIP: guinea pig anti-VIP-antibody + anti-guinea pig-IgG-antibody with Cy5
Figure 5	FG: rabbit anti-FG-antibody + TSA with Alexa Fluor 488 DAT (tdTomato): rat anti-RFP-antibody + anti-rat-IgG-antibody with Alexa Fluor 594 TH: mouse anti-TH-antibody + anti-mouse-IgG-antibody with Alexa Fluor 647
Figure 6	FG: rabbit anti-FG-antibody + TSA with Alexa Fluor 488 DAT (tdTomato): rat anti-RFP-antibody + anti-rat-IgG-antibody with Alexa Fluor 594 VIP: guinea pig anti-VIP-antibody + anti-guinea pig-IgG-antibody with Cy5
Figure 7	FG: rabbit anti-FG-antibody + TSA with Alexa Fluor 488 TH: mouse anti-TH-antibody + anti-mouse-IgG-antibody with Alexa Fluor 594 VIP: guinea pig anti-VIP-antibody + anti-guinea pig-IgG-antibody with Cy5
Figure 8	FG: rabbit anti-FG-antibody + TSA with Alexa Fluor 488 VGlut2 (tdTomato): rat anti-RFP-antibody + anti-rat-IgG-antibody with Alexa Fluor 594 VIP: guinea pig anti-VIP-antibody + anti-guinea pig-IgG-antibody with Cy5

Unless otherwise noted, fluorescence images were acquired using a laser scanning confocal microscope (LSM710, Carl Zeiss Microimaging, Jena, Germany). Alexa Fluor 488 was excited by a 488-nm Ar laser, and the emitted fluorescence was filtered with a 493- to 556-nm bandpass filter. Alexa Fluor 594 was excited by a 561 nm Diode Pumped Solid State laser, and the emitted fluorescence was filtered with a 589- to 628-nm bandpass filter. Alexa Fluor 647 and Cy5 were excited by a 633-nm He–Ne laser and emitted fluorescence was filtered with a 643- to 759-nm low-pass filter. Images of each dye were taken sequentially to avoid bleed-through artifacts. The fluorescence intensity levels were adjusted using FIJI (ImageJ, NIH, MD, USA). Brightfield images were acquired using a microscope (BZ-9000, Keyence, Osaka, Japan).

### The combination of fluorescent *in situ* hybridization and immunohistochemistry

Digoxigenin (DIG)-labeled antisense riboprobes were made from cDNAs of mouse DAT (nucleotides of 2–210, GenBank accession number: NM_010020.3). The specificity of the riboprobe was determined by the absence of staining in sections reacted with the sense riboprobe.

Two adult C57BL/6 mice were deeply anesthetized with isoflurane and perfused with 4% paraformaldehyde in 0.1 m PB (pH7.4). The brains were dissected out, postfixed, cryoprotected, and sectioned in the same manner shown above. The sections were washed in 0.1 m PB (pH 7.0) for 5 min twice, immersed in PB containing 0.3% Triton X-100, and rinsed in 0.1 m PB. Then, the sections were acetylated for 10 min at room temperature with 0.003% acetic acid anhydrate, 1.3% (v/v) triethanolamine, and 6.5% (w/v) HCl diluted in DEPC-treated water. After being rinsed twice in 0.1 m PB, the sections were incubated for 1 h at 70°C in a prehybridization buffer containing 50% (v/v) formamide, 5 × SSC buffer (a 5× concentration of SSC buffer containing 16.65 mm sodium chloride and 16.65 mm sodium citrate buffer, pH 7.0), 2% blocking reagents (Roche Diagnostics, Mannheim, Germany), 0.1% N-lauroylsarcosine (NLS), and 0.1% sodium dodecyl sulfate. Sections were hybridized with 1 μg/ml DIG-labeled cRNA probe for DAT in freshly prepared prehybridization buffer for 20 h at 63.5°C. After two washes in 2 × SSC, 50% formamide, and 0.1% NLS for 20 min at 60°C, the sections were rinsed in 2 × SSC with 0.1% NLS for 20 min twice at 37°C, and in 0.2 × SSC with 0.1% NLS for 20 min twice at 37°C. These sections were incubated with 1% blocking reagent (Roche) diluted in Tris–HCl (pH 7.5) and 0.15 m NaCl (TS7.5) for 1 h at room temperature and then with alkaline phosphatase-conjugated sheep anti-DIG antibody Fab fragment (1:2000; Roche), and guinea-pig anti-VIP (1:200) in 1% blocking reagent (Roche) diluted in TS7.5 at room temperature overnight. The sections were rinsed three times and incubated with Cy5-conjugated donkey anti-guinea-pig IgG (1:200; Jackson ImmunoResearch) in 1% blocking reagent (Roche) diluted in TS7.5 for 1 h. Finally, to visualize bound alkaline phosphatase, sections were developed with 0.005% (w/v) 4-chloro-2-methylbenzenediazonium hemi-zinc chloride (Fast Red TR, Roche), 1% (v/v) 2-hydroxy-3-naphtoic acid-2′-phenylanilide phosphate (Roche) diluted in 0.1 m Tris–HCl (pH 8.0), 0.15 m NaCl, and 10 mm MgCl_2_ for 30 min at room temperature. The sections were mounted on glass slides with CC/Mount (DBS).

### Data analysis

FIJI (ImageJ, NIH) was used to quantify the fiber signal intensity and normalized fluorescence intensity. The fiber signal intensity determined here was the subtraction of the background signal intensities from the signal intensity in each region. The normalized fluorescence intensity determined here was the fluorescence intensity in the lateral part of CeA (CeL) or the oval nucleus of BNST (BNST_OV)_ divided by the fluorescence intensity in the striatum in the same section. These intensities for each region were sampled from the right hemisphere of each subject. Data are expressed as means ± SEM. Permutation *t* tests followed by Holm’s correction were used for the multiple comparisons and permutation *t* tests were used to compare averages. Shapiro-Wilk test was used to assess the normality of distributions. Statistical significance was set at *p* < 0.05. Mean, *p*-value, confidence interval (CI), and some panels were calculated and generated by using the web application of Estimation stats (Ho et al., 2019). Information for the primary statistical analysis is provided in Table 2.

**Table 2:** Statistical table

	Data structure	Type of test	Power
a	Normal distribution	Permuted *t* test followed by Holm’s correction	[95.0%CI −54.8, −38.8]
b	Normal distribution	Permuted *t* test followed by Holm’s correction	[95%CI −61.0, −40.3]
c	Normal distribution	Permuted *t* test followed by Holm’s correction	[95.0%CI −84.1, −53.2]
d	Normal distribution	Permuted *t* test followed by Holm’s correction	[95.0%CI −99.0, −56.5]
e	Normal distribution	Permuted *t* test followed by Holm’s correction	[95.0%CI −1.86, 32.4]
f	Normal distribution	Permuted *t* test followed by Holm’s correction	[95.0%CI −8.39, −0.933]
g	Normal distribution	Permuted *t* test	[95.0%CI −1.67, −1.31]
h	Normal distribution	Permuted *t* test	[95.0%CI −1.71, −1.1]

## Results

### DAT neurons in the DR-PAG region consist of heterogeneous subpopulations

**Figure 1.**
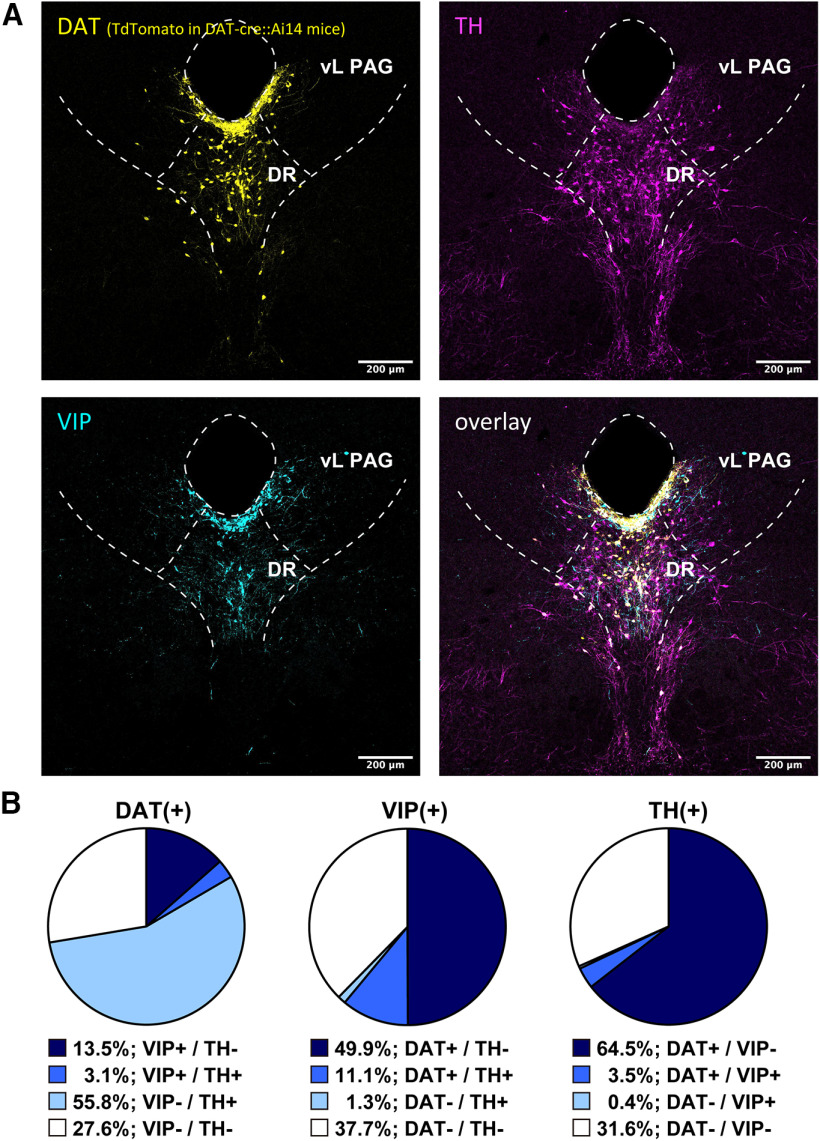
DAT^DR-PAG^ neurons are composed of at least two subpopulations. ***A***, Representative fluorescent images of each marker in the DR-PAG region. Top left, DAT signal was visualized with anti-RFP antibody in DAT-Cre::Ai14 mice (yellow). Top right, TH signal was visualized with anti-TH antibody (magenta). Bottom left, VIP signal was visualized with anti-VIP antibody (cyan). Bottom right, Three signals were overlaid. TH and VIP signals were rarely overlapped. ***B***, Pie charts showing the composition of each marker in the DR-PAG region. The percentages are calculated from four DAT-Cre::Ai14 mice.

**Figure 2.**
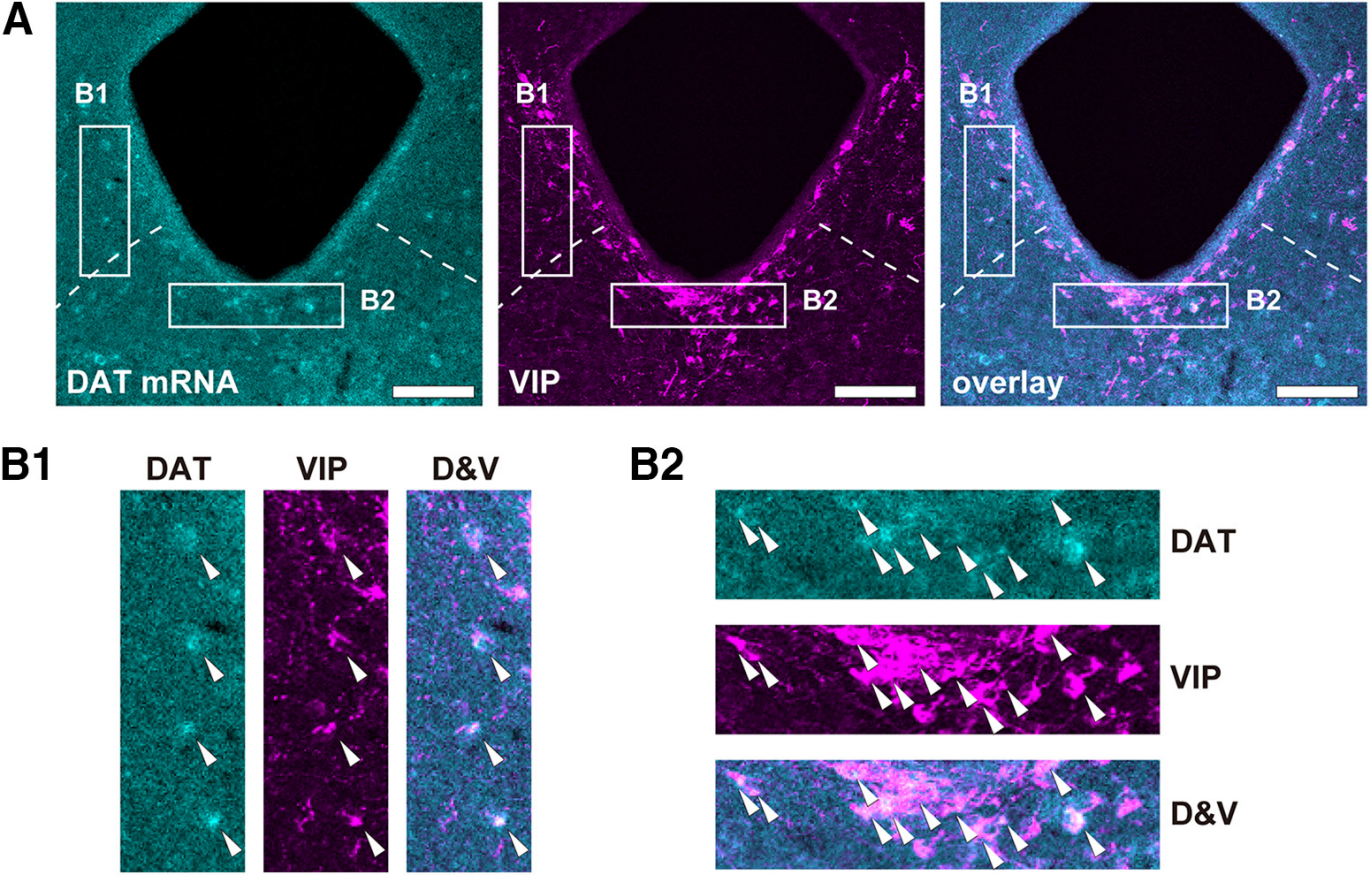
VIP^DR-PAG^ neurons are DAT-mRNA-positive. ***A***, Left, DAT signal was visualized with DAT *in situ* hybridization (cyan). Middle, VIP signal was visualized with anti-VIP antibody (magenta). Left, Two signals were overlaid. ***B***, B1 and B2 show high-magnification images corresponding to rectangle areas B1 and B2 in panel ***A***. White arrow heads indicate DAT and VIP double positive cell bodies. D&V represent overlaying images of DAT and VIP signals. DAT and VIP signals were colocalized in a number of neurons.

First, we examined the expression of DAT in the DR-PAG region (DAT^DR-PAG^), depending on the expression of tdTomato of DAT-cre::Ai14 mice. As shown in Figure 1*A*, the presence of DAT-positive cell bodies was confirmed in the DR-PAG region from the ventral PAG to the linear nucleus. This is consistent with previous reports that described the presence of DA neurons in this region (Li et al., 2016; Matthews et al., 2016; Cho et al., 2017; Groessl et al., 2018). With anti-TH and anti-VIP antibodies, the TH-positive and VIP-positive cell-bodies were also confirmed, as reported previously (Fig. 1*A*; Hioki et al., 2010; Dougalis et al., 2012; Poulin et al., 2014). TH-positive cell bodies were located in the DR and linear nucleus regions. Most VIP-positive cell bodies were located in the ventral part of the periaqueductal region. Next, we examined the colocalization of DAT, TH, and VIP immunostaining in this region. The majority of the DAT^DR-PAG^ neurons colocalized with TH or VIP signals (Fig. 1*A*,*B*; DAT+/TH+ 59.9%, DAT+/VIP+ 16.6%, and DAT+/TH+/VIP+ 3.1% of the all DAT^DR-PAG^ neurons; from four animals), only a few populations of DAT^DR-PAG^ neurons were labeled with both TH and VIP. This result clearly indicates that the DAT^DR-PAG^ neurons are heterogeneous. We examined the co-labeling of these three signals (DAT, TH, and VIP) for TH+ cells and for VIP+ cells in this region as well. Among the TH+ cells, 68.0% were positive for the DAT signal and 3.9% were positive for the VIP signal (Fig. 1*B*; from four animals). Among the VIP+ cells, 61.0% were positive for the DAT signal and 12.4% were positive for the TH signal (Fig. 1*B*; from four animals). It is commonly expected that DAT neurons also express TH, however, there is a significant portion of nonoverlapping of DAT-positive and TH-positive neurons. A possibility then arises that some off-target expressions of Cre-recombinase were introduced in VIP+/TH– neurons in DAT-Cre mice. To exclude this possibility, we also examined the colocalization of VIP immunoreactivity and DAT mRNA. Among the VIP+ neurons in the periaqueductal area, 53.6% were co-labeled with DAT *in situ* hybridization signal (Fig. 2 from two animals). Given that the sensitivity of *in situ* hybridization is regarded to be slightly lower than that of the Cre-loxP system, this result is likely to rule out the possibility of cre-recombinase off-target expression and support the idea that there is a certain population of DAT^DR-PAG^ neurons that express VIP but not TH.

### The main target of DAT^DR-PAG^ neurons is CeL and BNST_OV_

**Figure 3.**
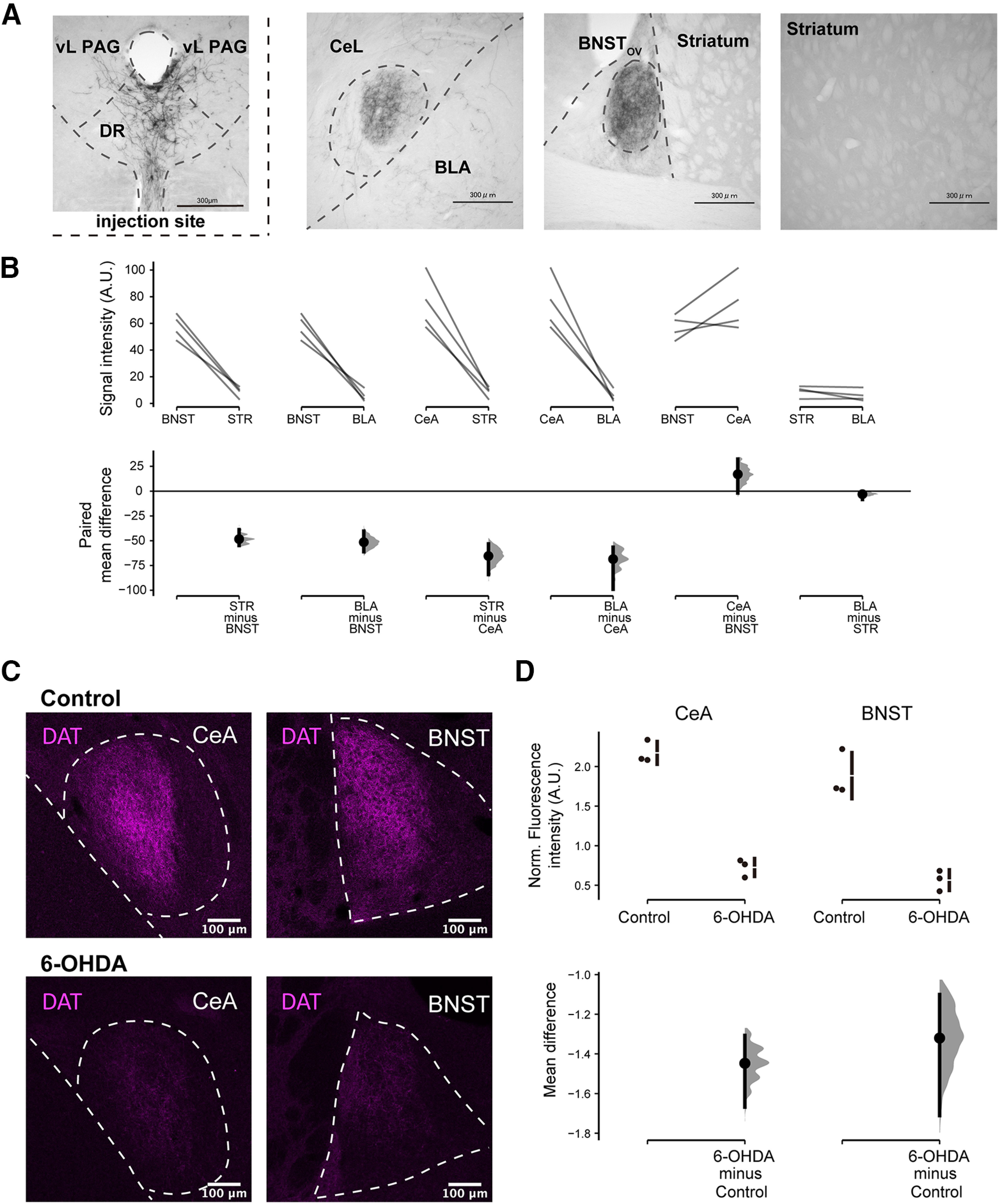
DAT^DR-PAG^ neurons innervate exclusively onto the CeL and BNST_OV_, and a vast majority of DAT fibers in these regions originated from the DR-PAG. ***A***, Representative images of AAV5-EF1α-DIO-hChR2-EYFP injection onto the DR-PAG region of a DAT-Cre mouse. EYFP signal was visualized with anti-GFP antibody following DAB staining. Top left, Injection site of the AAV onto a DAT-Cre mouse. Many DAT+ neurons were observed in this region. Top middle left, Fiber terminals were restricted in the CeL. Top middle right, Fiber terminals were restricted in the BNST_OV_. Top right, No fiber was observed in the striatum. ***B***, Paired mean differences for six comparisons are shown in the Cumming estimation plot. Signal intensities from four DAT-Cre mice are plotted on the upper axes; each paired set of observations is connected by a line. On the lower axes, each paired mean difference is plotted as a bootstrap sampling distribution. Mean differences are depicted as dots; 95% CIs are indicated by the ends of the vertical error bars. The fiber signal intensities in the BNST_OV_ and CeL were significantly higher than those in other regions. ***C***, Comparison of fiber fluorescence intensities in the CeL and BNST_OV_ between control and 6-OHDA-injected mice. Control, In the CeL and BNST_OV_, dense DAT+ fibers were observed. 6-OHDA, In contrast, DAT+ fibers were barely observed. ***D***, Mean differences for two comparisons are shown in the Cumming estimation plot. Signal intensities from three DAT-Cre::Ai14 mice are plotted on the upper axes; each mean difference is plotted on the lower axes as a bootstrap sampling distribution. Mean differences are depicted as dots; 95% CIs are indicated by the ends of the vertical error bars. There were statistically significant differences between control and 6-OHDA-injected mice.

To study the innervation pattern of DAT^DR-PAG^ neurons, we specifically labeled DAT^DR-PAG^ neurons with EYFP by injecting AAV5-EF1α-DIO-hChR2-EYFP into the DR-PAG region of DAT-Cre mice. Axonal fibers labeled with anti-GFP antibodies were located densely in the BNST_OV_ and CeL. In contrast, fibers labeled were rarely observed in the striatum and basolateral amygdala (BLA), despite that these regions are known to receive dense DA innervation (Fig. 3*A*). The fiber signal intensities for the BNST_OV_, CeL, striatum, and BLA were 57.5 ± 4.5, 74.5 ± 9.9, 9.1 ± 2.1, and 6.0 ± 2.2 (Fig. 3*B*; arbitrary units; quantified from four animals), respectively. The fiber densities in the BNST_OV_ and CeL were significantly higher than those in the other regions (Fig. 3*B*; permutation *t* test followed by Holm’s correction; BNST_OV_ versus striatum and BLA were statistically significant, *p* < 0.001^a^ and 0.001^b^, respectively, nominal thresholds = 0.0083 and 0.01, respectively; CeL versus striatum and BLA were statistically significant, *p* < 0.001^c^ and 0.001^d^, respectively; nominal thresholds = 0.0125 and 0.0167, respectively; for the other combinations *p* > 0.05^e,f^). We also tested the impact of DAT^DR-PAG^ neuron ablations on the DAT signals in the CeL and BNST_OV_. For this purpose, we injected 6-OHDA into the DR-PAG region. 6-OHDA is known to ablate DA neurons (Breese and Traylor, 1972; Kirik et al., 1998; Blum et al., 2001). The intensities of DAT+ fiber fluorescence in the CeL and BNST_OV_ were remarkably decreased by 6-OHDA injection, compared with the naive control case (Fig. 3*C*,*D*; normalized fluorescence intensity, arbitrary units; CeL, control 2.17 ± 0.08 vs 6-OHDA 0.73 ± 0.07, permutation *t* test, *p* < 0.001^g^; BNST_OV_, control 1.88 ± 0.17 vs 6-OHDA 0.56 ± 0.08, permutation *t* test, *p* < 0.001^h^; three animals for each group). These results indicate that the DAT^DR-PAG^ neurons are the primary sources of DAT innervation in the CeL and BNST_OV_.

### Properties of DR-PAG neurons that have projections to CeA or BNST

**Figure 4.**
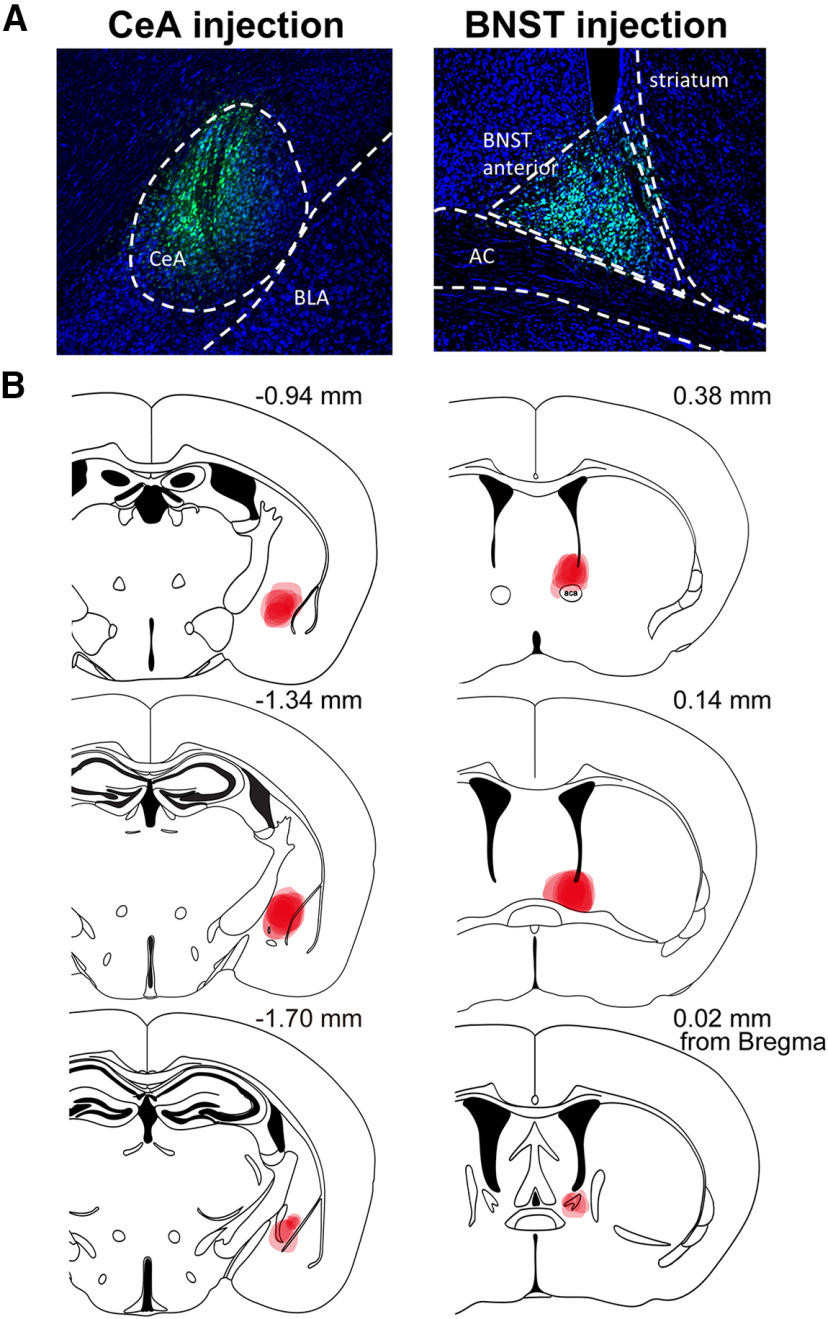
Summaries of the FG injection sites. ***A***, Representative images of the FG injections into the CeA and BNST. The green signal indicates the FG signal visualized with an anti-FG antibody. The blue background signal shows fluorescent Nissl staining with PI. ***B***, FG-injected areas are shown in the atlas for the CeA and BNST. Bold red indicates the areas commonly covered in the majority of subjects. Dim red indicates the area covered by injection at least in one animal. Atlas figures were modified from Franklin and Paxinos (2007). The distances from the bregma are indicated at the top right of each atlas.

**Figure 5.**
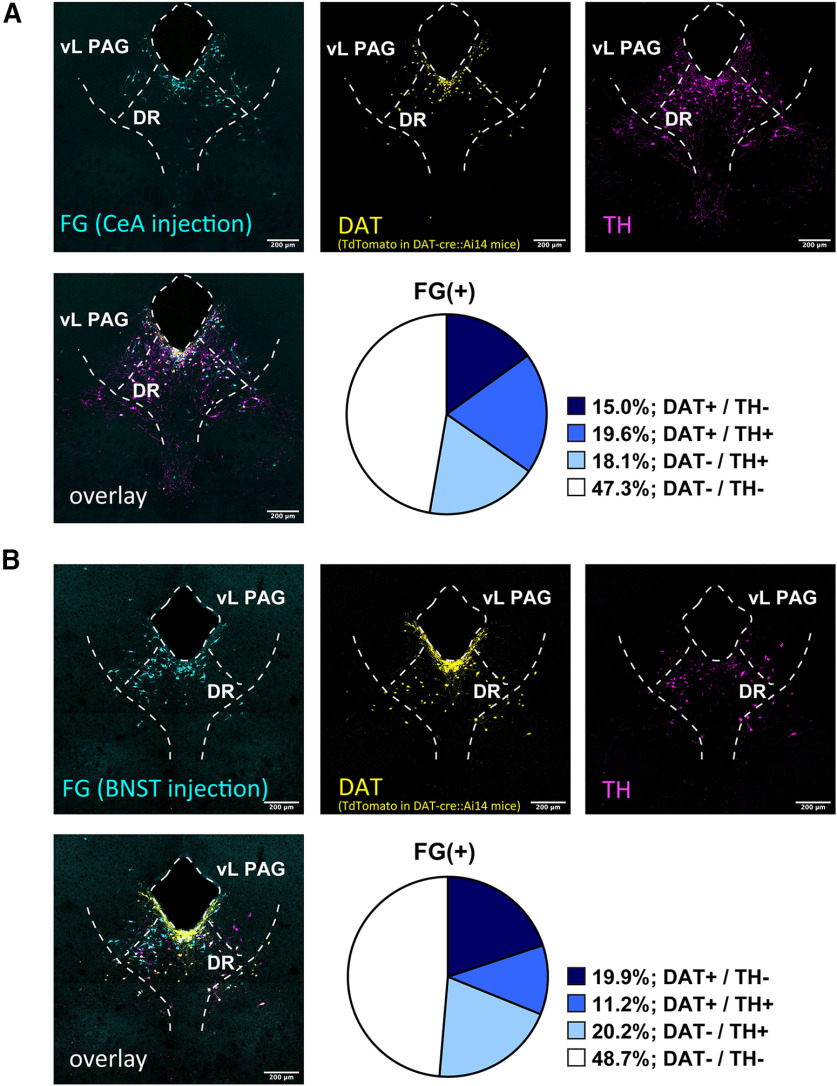
FG retrograde tracing revealed that DAT+/TH+ neurons similarly innervate the CeA and BNST. ***A***, Representative fluorescent images of each marker in the DR-PAG region. Top left, FG signal was visualized with anti-FG antibody in DAT-cre::Ai14 mice (cyan). Top middle, DAT signal was visualized with anti-RFP antibody (yellow). Top right, TH signal was visualized with anti-TH antibody (magenta). Bottom left, Three signals were overlaid. Bottom left, The pie chart indicates the composition of FG+ neurons (CeA injection) in the DR-PAG region. ***B***, Representative fluorescent images of each marker in the DR-PAG region. Top left, FG signal was visualized with anti-FG antibody in DAT-cre::Ai14 mice (cyan). Top middle, DAT signal was visualized with anti-RFP antibody (yellow). Top right, TH signal was visualized with anti-TH antibody (magenta). Bottom left, Three signals were overlaid. Bottom left, The pie chart indicates the composition of FG+ neurons (BNST injection) in the DR-PAG region.

**Figure 6.**
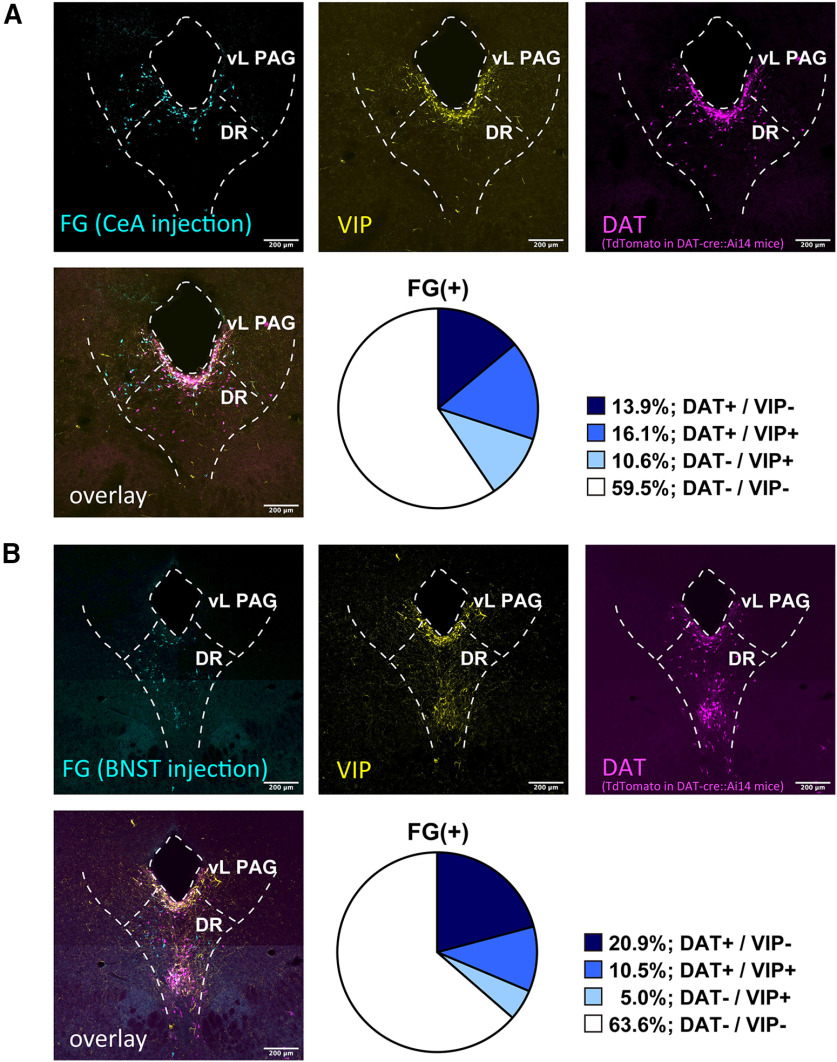
FG retrograde tracing revealed that the DAT+/VIP+ neurons similarly innervate the CeA and BNST. ***A***, Representative fluorescent images of each marker in the DR-PAG region. Top left, FG signal was visualized with anti-FG antibody in DAT-cre::Ai14 mice (cyan). Top middle, VIP signal was visualized with anti-VIP antibody (yellow). Top right, DAT signal was visualized with anti-DAT antibody (magenta). Bottom left, Three signals were overlaid. Bottom right, The pie chart indicates the composition of the FG+ neurons (CeA injection) in the DR-PAG region. ***B***, Representative fluorescent images of each marker in the DR-PAG region. Top left, FG signal was visualized with anti-FG antibody in DAT-cre::Ai14 mice (cyan). Top middle, VIP signal was visualized with anti-VIP antibody (yellow). Top right, DAT signal was visualized with anti-DAT antibody (magenta). Bottom left, Three signals were overlaid. Bottom right, The pie chart indicates the composition of the FG+ neurons (BNST injection) in the DR-PAG region.

**Figure 7.**
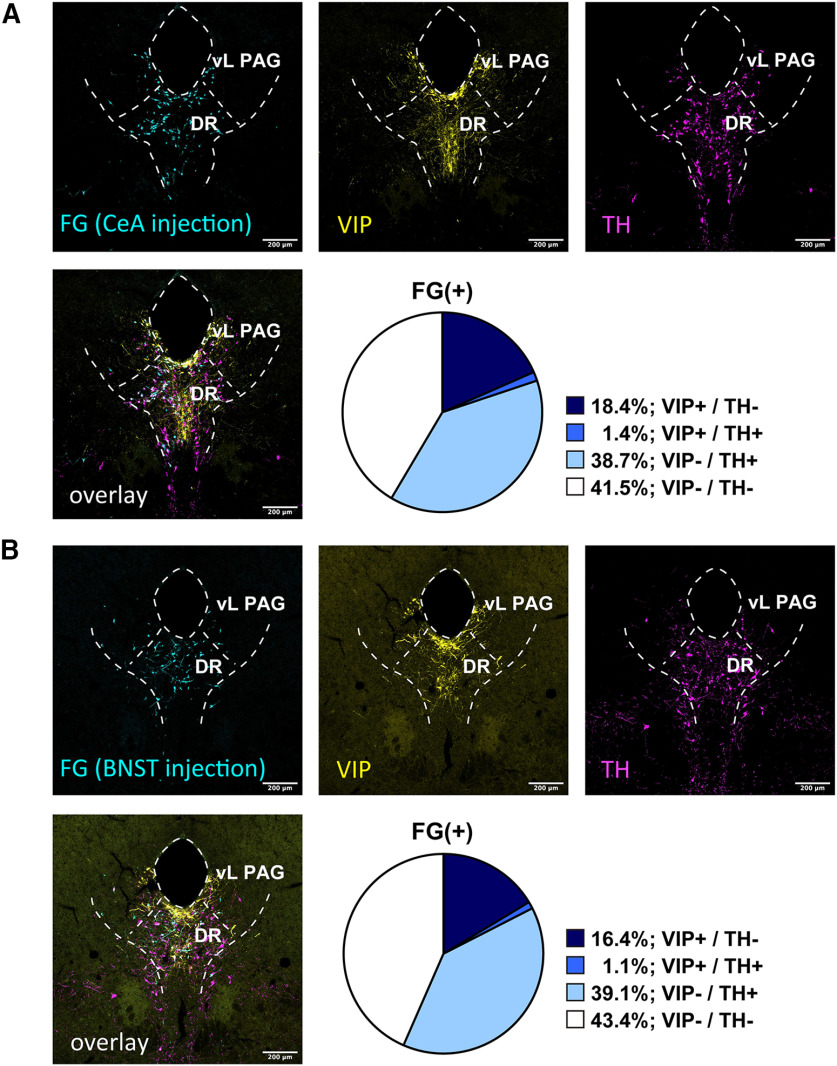
FG retrograde tracing revealed that the TH+ or VIP+ neurons similarly innervate the CeA and BNST. ***A***, Representative fluorescent images of each marker in the DR-PAG region. Top left, FG signal was visualized with anti-FG antibody (cyan). Top middle, VIP signal was visualized with anti-VIP antibody (yellow). Top right, TH signal was visualized with anti-TH antibody (magenta). Bottom left, Three signals were overlaid. Bottom right, The pie chart indicates the composition of the FG+ neurons (CeA injection) in the DR-PAG region. ***B***, Representative fluorescent images of each marker in the DR-PAG region. Top left, FG signal was visualized with anti-FG antibody in DAT-cre::Ai14 mice (cyan). Top middle, VIP signal was visualized with anti-VIP antibody (yellow). Top right, TH signal was visualized with anti-TH antibody (magenta). Bottom left, Three signals were overlaid. Bottom right, The pie chart indicates the composition of the FG+ neurons (BNST injection) in the DR-PAG region.

To retrogradely label the cell body of DR-PAG neurons projecting onto the extended amygdala, we made restricted small injections of FG centered in CeL or BNST_OV_. We investigated the colocalization of signals of FG, DAT (depending on tdTomato expression), and TH or VIP in DAT-Cre::Ai14 mice. The summary of injection sites for the CeA and BNST is shown in Figure 4. First, we examined the colocalization of FG, DAT, and TH, by injecting FG into the CeA or BNST. After FG injection into the CeA, 34.6% of the CeA projecting neurons were DAT-positive and 37.7% of the neurons were TH-positive (Fig. 5*A*; from three animals). Additionally, 19.6% of the retrogradely labeled neurons were positive for both DAT and TH (FG/DAT/TH; Fig. 5*A*; from three animals). Similar to this, after FG injection into the BNST, 31.1% of the BNST projecting neurons were DAT-positive and 31.4% of the neurons were TH-positive (Fig. 5*B*; from three animals). Additionally, 11.2% of the retrogradely labeled neurons were positive for both DAT and TH (FG/DAT/TH; Fig. 5*B*; from three animals). Next, we examined the colocalization of FG, DAT, and VIP. After FG injection into the CeA, 30.0% of the CeA projecting neurons were DAT-positive and 26.7% of the neurons were VIP-positive (Fig. 6*A*; from three animals). Additionally, 16.1% of retrogradely labeled neurons were positive for both DAT and TH (FG/DAT/TH; Fig. 6*A*; from three animals). Among the VIP+ neurons innervating the CeA, 60.3% were DAT+ neurons. Similar to these results, after FG injection into the BNST, 31.4% of the BNST projecting neurons were DAT-positive and 15.5% of the neurons were VIP-positive (Fig. 6*B*; from three animals). Additionally, 10.5% of retrogradely labeled neurons were positive for both DAT and VIP (FG/DAT/VIP; Fig. 6*B*; from three animals). Among the VIP+ neurons innervating the BNST, 67.7% were DAT+ neurons. We also tested the colocalization of immunolabeled signals of FG, TH, and VIP. After FG injection into the CeA, 40.1% of retrogradely labeled neurons were positive for TH, 19.8% of the neurons were positive for VIP, and 1.4% of the neurons were positive for both TH and VIP (FG/TH/VIP; Fig. 7*A*; from four animals). After FG injection into the BNST, 40.2% of retrogradely labeled neurons were positive for TH, 17.3% of the neurons were positive for VIP, and 1.1% of the neurons were positive for both TH and VIP (FG/TH/VIP; Fig. 7*B*; from four animals). Altogether, the two subpopulations of DAT^DR-PAG^ neurons, which are DAT/TH and DAT/VIP, had similar projection patterns onto the CeA and BNST.

### The majority of VIP^DR-PAG^ neurons that have projections onto the CeA or BNST are glutamatergic

**Figure 8.**
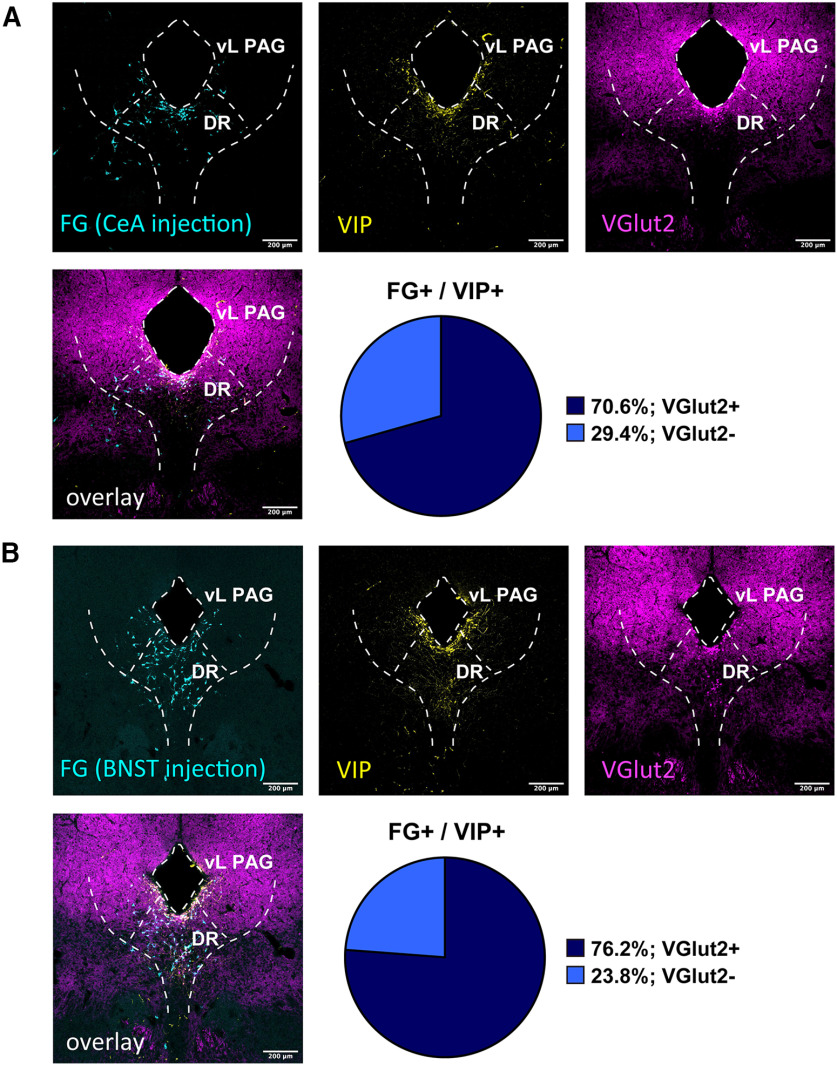
FG retrograde tracing revealed that the VIP+/VGlut2+ neurons similarly innervate the CeA and BNST. ***A***, Representative fluorescent images of each marker in the DR-PAG region. Top left, FG signal was visualized with anti-FG antibody in VGlut2-cre::Ai14 mice (cyan). Top middle, VIP signal was visualized with anti-VIP antibody (yellow). Top right, VGlut-2 signal was visualized with anti-RFP antibody (magenta). Bottom left, Three signals were overlaid. Bottom right, The pie chart indicates the composition of FG+/VIP+ neurons (CeA injection) in the DR-PAG region. ***B***, Representative fluorescent images of each marker in the DR-PAG region. Top left, FG signal was visualized with anti-FG antibody in VGlut2-Cre::Ai14 mice (cyan). Top middle, VIP signal was visualized with anti-VIP antibody (yellow). Top right, VGlut2 signal was visualized with anti-RFP antibody (magenta). Bottom left, Three signals were overlaid. Bottom right, The pie chart indicates the composition of FG+/VIP+ neurons (BNST injection) in the DR-PAG region.

It has been suggested that the DA^DR-PAG^ neurons corelease glutamate as well as dopamine (Matthews et al., 2016; Groessl et al., 2018). Since we have found that DAT^DR-PAG^ neurons consisted of two subpopulations, which are TH–/VIP+ and TH+/VIP–, here comes a question whether both subpopulations are glutamatergic. To address this question, we examined the colocalization of FG (injected into the CeA or BNST), VIP, and VGlut2 (depending on tdTomato expression) signals in VGlut2-Cre::Ai14 mice, after an injection of FG into the CeA or BNST. After FG injection into the CeA, 70.6% of the FG/VIP double positive neurons were positive for VGlut2 signal (Fig. 8*A*; three animals). After FG injection into the BNST, 76.2% of the FG/VIP double positive neurons were positive for VGlut2 signal (Fig. 8*B*; three animals). These results suggested that the majority of VIP^DR-PAG^ neurons projecting to the BNST/CeA are glutamatergic. We also tried to examine whether TH neurons in the DR-PAG region are VGlut2-positive or not in VGlut2-cre::Ai14 mice. However, the expression of tdTomato in the DR-PAG region, except the periaqueductal area where the majority of VIP neurons were observed, was not strong enough to quantify the colocalization of TH and VGlut2. At least, we confirmed that the majority of VIP^DR-PAG^ neurons could be glutamatergic, innervating the CeA and BNST similarly. From the results shown in Figures 1, 2, and 6, these VIP^DR-PAG^ neurons are likely to be DAT+/VIP+ neurons. Our experiment did not exclude the possibility that some TH^DR-PAG^ neurons were also glutamatergic.

### The extent of DAT expression in DAT^DR-PAG^ neurons is relatively lower than that of DAT neurons in the ventral tegmental area (VTA) and the substantia nigra compacta (SNc)

**Figure 9.**
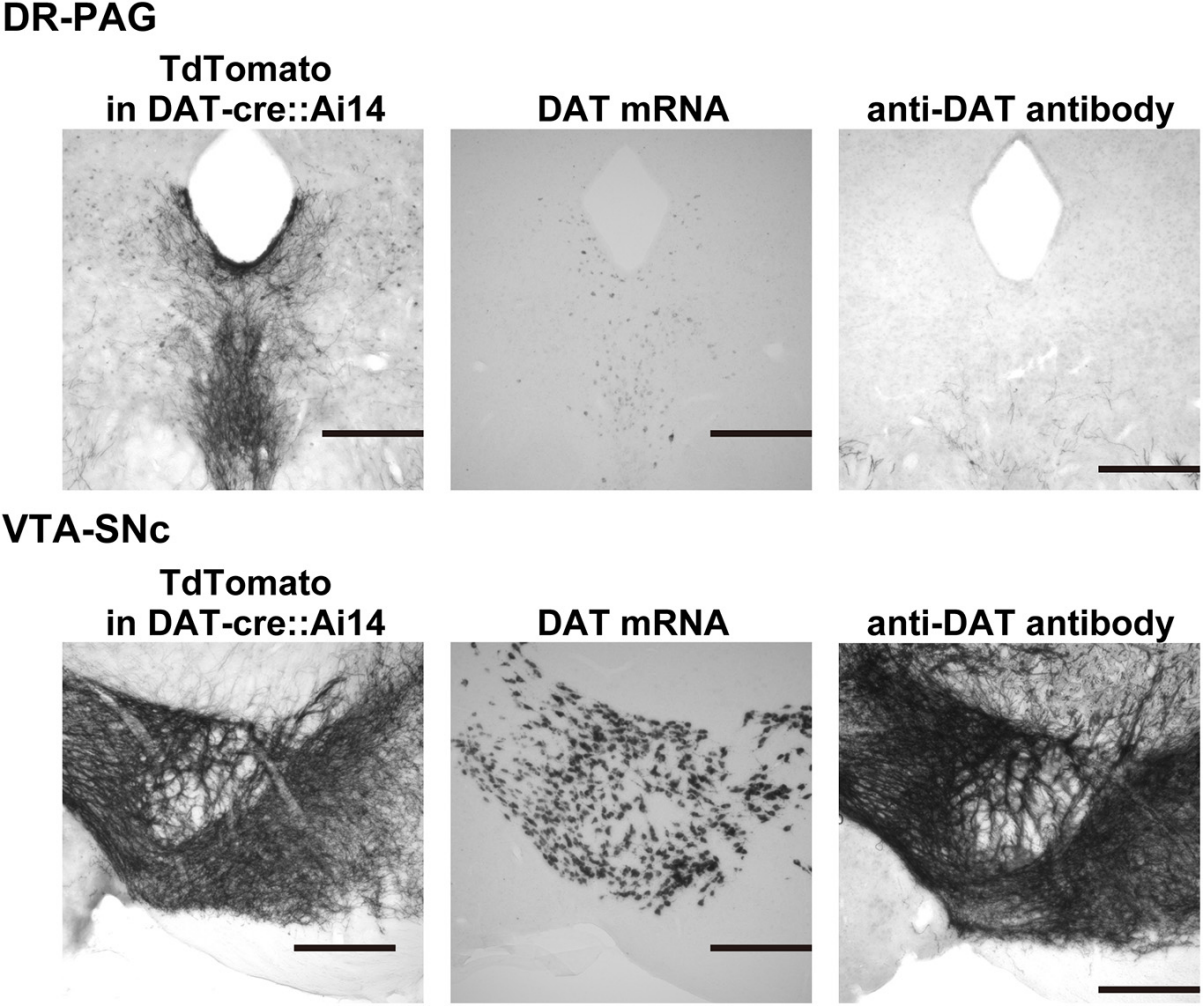
The extent of DAT protein expression is lower in the DAT^DR-PAG^ neurons. DR-PAG, Left, Staining of DAT neurons in a DAT-cre::Ai14 mouse. Many cell bodies and fibers are confirmed. Middle, With DAT *in situ* hybridization, dim cell bodies were confirmed in the DR-PAG region. Right, Immunostaining of DAT barely stained cell bodies in the DR-PAG region. VTA-SNc, In all three methods, strong DAT signals were confirmed. Each black scale bar indicates 300 μm.

As already shown, many DAT-positive neurons exist in the DR-PAG region. This was confirmed in DAT-Cre::Ai14 mice (Figs. 1, 5, 6, 9) and by RNA *in situ* hybridization (Figs. 2, 9). However, the number of DAT-antibody-positive cell-bodies in DR-PAG was obviously lower than that of the DAT-mRNA-positive cell-bodies or tdTomato-positive cell-bodies in DAT-Cre::Ai14 mice (Fig. 9; DR-PAG). In contrast, a large number of DAT-antibody-positive cell-bodies were observed in the VTA and SNc regions, which was similar to that confirmed in DAT *in situ* hybridization and in DAT-cre::Ai14 mice (Fig. 9; VTA-SNc). Furthermore, the DAT-mRNA signal in the DR-PAG was less bold than that in the VTA-SNc. These results suggest that the DAT protein translation is much lower in DAT^DR-PAG^ neurons than in DAT^VTA-SNc^ neurons, which may reflect the different roles of these DAT neuron populations.

## Discussion

In this study, we confirmed that DAT^DR-PAG^ neurons consist of the following two subpopulations: DAT+/TH+/VIP– and DAT+/TH–/VIP+ in male mice. These two populations similarly innervate the CeL and BNST_OV_, which are the main targets of DAT^DR-PAG^ neurons. Furthermore, the majority of VIP^DR-PAG^ neurons projecting onto the CeA and BNST are suggested to be glutamatergic. This is the first study that identified two different subpopulations of DAT neurons in the DR-PAG region. These results implied possible functional heterogeneity among DAT^DR-PAG^ neurons.

### DAT^DR-PAG^ neurons consist of two subpopulations

Consistent with previous reports (Poulin et al., 2018; Cardozo Pinto et al., 2019; Lin et al., 2020), we also confirmed that many DAT+ neurons exist in the DR-PAG region using DAT-Cre::Ai14 mice, DAT *in situ* hybridization, and anti-DAT-antibody staining. However, these DAT neurons were different from typical DAT neurons, such as DAT neurons in the VTA or SNc, since the protein expression of DAT at soma was much lower as shown in Figure 9. This result suggests that DAT^DR-PAG^ neurons may have different functional characteristics from other DAT-positive neurons.

Since DAT is regarded as a reliable marker for DA neurons (Augood et al., 1993; Ciliax et al., 1999; Lewis et al., 2001), we first expected that basically all tdTomato+ neurons in DAT-Cre::Ai14 mice were TH-positive dopaminergic neurons. Indeed, more than half of tdTomato+ DAT^DR-PAG^ neurons were TH-positive. However, a substantial amount of the DAT^DR-PAG^ neurons, especially those located in the periaqueductal region, were TH-negative and VIP-positive. Previous studies focusing on mRNA expression have reported that all DAT, TH, and VIP mRNA were expressed in a single DAT^DR-PAG^ neuron (Poulin et al., 2014, 2018; La Manno et al., 2016; Hook et al., 2018; Tiklová et al., 2019). Also, TH and VIP signals were co-localized in the DR-PAG region in a study using TH-GFP mice (Dougalis et al., 2012). Our present findings, which are incompatible with these previous studies, would arise from the difference between mRNA and protein expression levels in these DAT^DR-PAG^ neurons. mRNA translation is subject to multiple modulatory factors, and mRNA existence does not ensure a high level of protein expression. Consistent with our results, a study showed that the tdTomato+ neurons located in the periaqueductal region in DAT-Cre::Ai14 were TH-negative (Cardozo Pinto et al., 2019; Kramer et al., 2021). We ruled out the off-target expression of Cre-recombinase in DAT-Cre mice, showing that these TH-negative neurons were VIP neurons expressing DAT mRNA. Therefore, it was concluded that DAT^DR-PAG^ neurons consist of two different subpopulations, which are DAT+/TH+/VIP– and DAT+/TH–/VIP+.

As we have demonstrated, DAT^DR-PAG^ neurons expressed lower amounts of DAT than other typical DAT neurons. Hence, the DAT+/TH+ subpopulation of DAT^DR-PAG^ neurons could be DA neurons with a feature different from that of other DA neurons. Subpopulations of DA neurons in the VTA, which innervate the prefrontal cortex, BLA, and a part of the nucleus accumbens, express a lower amount of DAT than the DA neurons innervating the striatum (Lammel et al., 2008). Given that the DR-PAG TH+ neurons are regarded as a subgroup of the A10 cluster (Trulson et al., 1985; Descarries et al., 1986), DAT+/TH+ neurons in the DR-PAG might have similar characteristics to the mesocorticolimbic DA neurons described in the previous report (Lammel et al., 2008). Additionally, TH-positive DA neurons in the hypothalamus (including A11) barely express DAT (Barraud et al., 2010; Koblinger et al., 2014; Zhang et al., 2021). These DA neurons with low DAT expression should comprise a distinct subpopulation assigned to different functions than the canonical DA neurons. We also confirmed the presence of the DAT+/TH–/VIP+ group in the DR-PAG region. Since TH expression seems to be lower in this group, these DAT+/TH–/VIP+ neurons will not act as DA neurons. In addition, we have found this population is VGlut2-positive. Thus, DAT+/TH–/VIP+ neurons will use glutamate rather than DA as the primary neurotransmitter. DAT+/TH– neurons have been also reported in various brain regions. In the A11 cluster, DAT+ fibers lacking TH were confirmed, even those were a tiny population (Koblinger et al., 2014). DAT+ neurons, lacking TH, in the ventral premammillary nucleus of the hypothalamus were glutamatergic (Soden et al., 2016). The noncanonical DAT neurons described in the present study might share similar functions with these DAT+/TH– neurons.

### The projection of DAT^DR-PAG^ neurons onto the extended amygdala

Using FG retrograde labeling, we confirmed that DAT^DR-PAG^ neurons innervate the CeA and BNST. Anterograde tracings and 6-OHDA lesion study revealed that DAT^DR-PAG^ neurons exclusively innervate the extended amygdala, especially the CeL and BNST_OV_. Consistent with these results, recent studies taking advantage of Cre-driver lines demonstrated that TH or DAT neurons in the DR-PAG region have projections onto the CeA and BNST (Matthews et al., 2016; Groessl et al., 2018; Poulin et al., 2018; Lin et al., 2020). In addition, using FG retrograde labeling similar to the present study, it was reported that many TH neurons in the DR-PAG region have projections onto the extended amygdala (Hasue and Shammah-Lagnado, 2002). In the study by Hasue and Shammah-Lagnado, many retrogradely labeled TH neurons were observed not only in the DR-PAG region but also in other TH clusters such as A9 or VTA, while in our current study, the vast majority of TH-positive retrogradely labeled neurons were restricted in the DR-PAG region. We assume that this difference stemmed from the difference in the amount of FG injected. Our injection was small and well restricted in the CeA and BNST compared with the study by Hasue and Shammah-Lagnado (2002). Also, our results suggested that the DAT+/TH–/VIP+ subpopulation would have glutamatergic projections onto the CeA and BNST. Consistent with this, VIP innervations from the DR-PAG region to the BNST, which was not specified as DAT+/VGlut2+, have been reported in several studies (Petit et al., 1995; Kozicz et al., 1998). This VIP signal in the BNST exhibits sexual dimorphism in humans, finches, and tree shrews (Zhou et al., 1995; Chung et al., 2002; Goodson et al., 2006; Ni et al., 2022). Yu et al. (2021) reported that the manipulation of DA^DR-PAG^ neuronal activities induced different behavioral modulations between male and female mice, which might be because of the sexual dimorphism of VIP fibers in BNST, originating from the VIP subpopulation of DA^DR-PAG^ neurons.

Adding to these accumulating reports, the present study confirmed that the two subpopulations of DAT^DR-PAG^ neurons, TH+/VIP– and TH–/VIP+, similarly innervate the CeA and BNST. According to the spatial distribution of retrogradely labeled neurons, both subpopulations of DAT^DR-PAG^ neurons seemed to bilaterally innervate the CeA and BNST. Consistent with this idea, single neuron tracing experiments demonstrated the existence of DAT^DR-PAG^ neurons innervating both the CeA and BNST (Lin et al., 2020). Thus, the possibility would arise that these innervations onto the CeA and BNST might have a broad impact on neural activities in both regions simultaneously. Additional experiments will be required to clarify this possibility in the future.

### Approaches to investigate physiological roles of DAT^DR-PAG^ neurons in various behaviors

Recently, many studies have investigated the functional importance of DA^DR-PAG^ neurons in various behaviors and emotional states; pain sensation (Li et al., 2016; Yu et al., 2021), social interactions (Matthews et al., 2016), fear learning (Groessl et al., 2018), responding to salient stimuli (Cho et al., 2017, 2021), and reward incentives (Lin et al., 2020, 2021). To study these functions, TH-Cre mice (Savitt et al., 2005) or DAT-Cre mice (Bäckman et al., 2006) were used to selectively manipulate DA neurons. However, as pointed out in previous reports, Cre-recombinase expression in TH-Cre mice is not well overlapping with TH protein expression in the DR-PAG region (Lammel et al., 2015; Stuber et al., 2015; Cardozo Pinto et al., 2019). In addition, it was implied that Cre-recombinase-positive neurons in TH-Cre mice involved more glutamatergic neurons than those of DAT-Cre mice (Cardozo Pinto et al., 2019). This characteristic of TH-Cre mice could be a part of the reason why some of the modulations on behaviors by DA^DR-PAG^ neurons reported in some studies were not confirmed in other studies. Thus, some studies on DA^DR-PAG^ neurons conducted with TH-Cre mice must be revisited using finer methods. Furthermore, a report has shown that some emotional behaviors related to anxiety are altered by VGlut2+ neurons in the DR-PAG region, not by DAT+ neurons (Taylor et al., 2019). Even in DAT-Cre mice, Cre-recombinase expression did not overlap well with TH neurons (Cardozo Pinto et al., 2019). Consistent with this report, we have found that a part of DAT^DR-PAG^ neurons is VIP neurons lacking TH in this study. Dissociation of the DAT and TH expression in the DR-PAG region was also reported by Kramer et al. (2021). We have shown, DAT^DR-PAG^ neurons consist of TH neurons and VIP/VGlut2 neurons, therefore, to elucidate the fine roles of DA neurons or DAT neurons in the DR-PAG region, precise genetic separation of neural subgroups must be required.

## Synthesis

Reviewing Editor: Mikko Airavaara, Helsingin yliopisto Decisions are customarily a result of the Reviewing Editor and the peer reviewers coming together and discussing their recommendations until a consensus is reached. When revisions are invited, a fact-based synthesis statement explaining their decision and outlining what is needed to prepare a revision will be listed below. The following reviewer(s) agreed to reveal their identity: Thomas Kash.

The authors describe the heterogeneous nature of DAT+ neurons in the DR/PAG and the nature of their projections to the extended amygdala. These insights are indeed valuable for those working with these neuron populations, but methodological concerns and lack of functional insight dampen enthusiasm for this manuscript. The reviewers found the study potentially interesting and suitable for publication if substantially revised.

1. First, little information is provided for the statistical analyses. It is unclear how distributions were assessed, and how power was calculated. Whether t-tests were paired or unpaired is not indicated, despite presumably sampling multiple regions from the same animals. It is also not indicated how N was determined for semi-quantitative studies of signal intensity (sampled from each hemisphere and averaged for each mouse? from only one hemisphere per mouse? each hemisphere used as a sample?). Furthermore, pay attention to the journal guidelines for presenting statistics. The use of estimation statistics is highly recommended. Also, statistics do not always support the interpretations of the authors. For example, comparisons of fiber intensity (e,f in table 2) are listed as non-significant but have an extremely low power of 0.03 or 0.05, suggesting the potential for a type II error.

2. Second, the only truly quantitative data in the manuscript is a measurement of fluorescence intensity signal, which needs to be more carefully controlled to yield meaningful results. Results shown in Figure 3B are subject to significant bias due to measuring antibody staining in separate brains without control regions. In order to interpet these results, the inclusion of a control region (STR??) is needed, which would be unchanged after the 6OHDA lesion if the authors hypothesis is correct.

3. Third, The figures can be improved for clarity.

-Some figures have helpful overlays of anatomical atlas regions (3, 4), while the rest do not. This should be kept the same throughout for ease of reading.

-Figure 2 asserts that VIP neurons in the DR-PAG co-localize with DAT mRNA (“DAT and VIP signals were colocalized in a number of neurons”). This cannot be appreciated from the micrographs provided. A second row of higher magnification images should be added, which clearly show DAT mRNA+ and - VIP neurons.

-The staining in the micrographs in Figures 5-8 is very difficult to see. The brightness of these images should be increased for publication.

4. Finally, the conclusions of the authors throughout are overstated considering the descriptive nature of these studies. For example, line 75-76 “These subpopulations of DAT DR-PAG neurons must play different roles in the regulation of affective behaviors.” No evidence in the manuscript directly supports these ideas.

5. Minor note : Line 269 there seems to be an error, it says neurons are positive for both DAT and TH but in parentheses (FG/DAT/VIP).

6. No data provided on how distributions were determined to be normal, as indicated on table 2. No information provided on how power was calculated, as indicated in table 2.

7. The authors only investigate male mice, this needs to be, minimally, justified.

8. Line 425, they claim that a manuscript from the stuber lab in 2015 identified similiar lack of TH Expression in the DR TH cre neurons. this is incorrect.

## Author Response

Thank you for your kind suggestions to improve this manuscript.

As detailed below, we have addressed all of the reviewer’s reservations.

The changes are highlighted in the marked text and “LXX” corresponds to lines in the marked text.

#1

Shapiro-Wilk test was used to assess whether the distribution is normal or not (L197).

Powers were calculated by using SPSS, however, powers were replaced with CIs in the current version of the manuscript following the guideline of eNeuro.

Intensities for each region were sampled from the right hemisphere of each subject. This explanation was added in the data analysis section (L193-194).

We used the estimation statics (permutation t-test) in this revised manuscript instead of paired or Welch’s t-test. These changes are reflected in the analysis section (L195-197), results (L244-247, L254-255), figure legends (L706-711, L715-719), figures, and table 2.

In the panels of Fig.3, we replaced the graphs with those reflecting estimation statics.

According to the low powers listed in the original table 2, to our understanding, the calculation of the power using “α = 0.05” for the statistically non-significant t-test basically provides a low power. Hence our interpretation of the results, “there was no statistical significance (we are not claiming that the means of two distributions are the same)”, would be still acceptable.

#2

To avoid the bias of different staining intensities between sections, we used the fluorescence intensity of the striatum in the section containing the CeA or BNST as the control signal. The normalized fluorescence intensity is calculated as the fluorescence intensity in the CeL or BNSTov divided by the fluorescence intensity in the striatum in the same section (L190-194).

#3

We added borderlines to clarify each region in all figs. For fig. 2, we added higher magnification images. Also, the brightness of Fig. 5-8 was increased.

#4

We made statements moderate (L21, L35, L76, L335, L367).

#5

We corrected it. Thank you for pointing this out (L276).

#6

Shapiro-Wilk test was used to assess whether the distribution is normal or not. Powers were calculated by using SPSS, however, powers are replaced with CIs. These explanations were added in the data-analysis section (L197-198).

#7

We made it clear that we have studied only male mice and this study focused on males. (L86-87, L330)

#8

Thank you for pointing out our mistake. We removed the ref. from this sentence (L433).
