# Erratum: Zhao et al., “Histochemical Characterization of the Dorsal Raphe-Periaqueductal Grey Dopamine Transporter Neurons Projecting to the Extended Amygdala”

**DOI:** 10.1523/ENEURO.0292-22.2022

**Published:** 2022-08-03

In the article, “Histochemical Characterization of the Dorsal Raphe-Periaqueductal Grey Dopamine Transporter Neurons Projecting to the Extended Amygdala,” by Qin Zhao, Tetsufumi Ito, Chika Soko, Yoshie Hori, Takafumi Furuyama, Hiroyuki Hioki, Kohtarou Konno, Miwako Yamasaki, Masahiko Watanabe, Satoshi Ohtsuka, Munenori Ono, Nobuo Kato, and Ryo Yamamoto, which published online on May 17, 2022, there were two errors.

The city name in affiliation two was misspelled; the correct affiliation should instead appear as “Systems Function and Morphology Laboratory, Graduate School of Innovative Life Science, University of Toyama, Toyama, Toyama 930–0194, Japan.”

On page 14, in the section *Approaches to investigate physiological roles of DAT^DR-PAG^ neurons in various behaviors*, Stuber et al., 2015 was incorrectly cited in line 13.

The online version has been corrected.

